# XHap: haplotype assembly using long-distance read correlations learned by transformers

**DOI:** 10.1093/bioadv/vbad169

**Published:** 2023-11-23

**Authors:** Shorya Consul, Ziqi Ke, Haris Vikalo

**Affiliations:** Department of Electrical and Computer Engineering, The University of Texas at Austin, Austin, TX 78712, United States; Department of Electrical and Computer Engineering, The University of Texas at Austin, Austin, TX 78712, United States; Department of Electrical and Computer Engineering, The University of Texas at Austin, Austin, TX 78712, United States

## Abstract

**Summary:**

Reconstructing haplotypes of an organism from a set of sequencing reads is a computationally challenging (NP-hard) problem. In reference-guided settings, at the core of haplotype assembly is the task of clustering reads according to their origin, i.e. grouping together reads that sample the same haplotype. Read length limitations and sequencing errors render this problem difficult even for diploids; the complexity of the problem grows with the ploidy of the organism. We present XHap, a novel method for haplotype assembly that aims to learn correlations between pairs of sequencing reads, including those that do not overlap but may be separated by large genomic distances, and utilize the learned correlations to assemble the haplotypes. This is accomplished by leveraging transformers, a powerful deep-learning technique that relies on the attention mechanism to discover dependencies between non-overlapping reads. Experiments on semi-experimental and real data demonstrate that the proposed method significantly outperforms state-of-the-art techniques in diploid and polyploid haplotype assembly tasks on both short and long sequencing reads.

**Availability and implementation:**

The code for XHap and the included experiments is available at https://github.com/shoryaconsul/XHap.

## 1 Introduction

Genetic variations between chromosomal copies of the DNA of a single individual eukaryotic organism, rooted in inherited or acquired mutations, have major implications on the organism’s cellular functions. A manifestation of genetic diversity in an individual’s genome is the variations between copies of chromosomes inherited from the individual’s parents. An ordered list of point mutations, i.e. single-nucleotide polymorphisms (SNPs), on an individual’s chromosomes is referred to as a haplotype. Haplotype information is of fundamental importance in a number of medical and pharmaceutical exploratory tasks. For instance, the presence of multiple variants at corresponding genes of homologous chromosomes could lead to different gene expression patterns which, in turn, may affect an individual’s susceptibility to diseases and response to therapeutic drugs (Tewhey *et al.* 2011). Besides this, haplotyping enables the identification of certain groups of SNP loci or compound heterozygosity that may be associated with diseases (Glusman *et al.* 2014). In addition to this, haplotype structure is used in non-invasive strategies for prenatal genetic diagnostics (Kitzman *et al.* 2012), and proven to be beneficial to the study of recombination patterns and gene identification under positive selection (Sabeti *et al.* 2002). This necessitates the reconstruction of haplotypes from sequencing data, otherwise known as the haplotype assembly problem.

While recent advancements in sequencing technologies have enabled routine studies of individual genetic blueprints, limitations of sequencing platforms render the haplotype assembly problem challenging. State-of-the-art sequencing technologies can be broadly organized into two categories according to the length of the reads that they generate. The high-throughput platforms, such as Illumina’s MiSeq and NovaSeq platforms, provide highly accurate (error rates ≈0.1%) but relatively short reads (typically, <500 bp). Such reads may enable highly accurate local reconstruction but these reads often cover an insufficient number of variants to fully phase haplotypes, resulting in haplotypes that are often fragmented into blocks that are generally difficult to phase. In contrast, most third generation sequencing platforms, including those by Pacific Biosciences (PacBio) and Oxford Nanopore Technologies (ONT), generate reads of much greater length (often longer than 10 kb) but typically suffer from higher error rates than short-read technologies (Wick *et al.* 2017). The long reads enable bridging across large genomic distances and thus aid with producing longer haplotypes and reducing the number of haplotype blocks; however, this benefit comes at the expense of generally inferior accuracy and higher cost-per-base as compared to the short reads provided by high-throughput platforms.

Regardless of the technology used to generate sequencing reads, at the core of haplotype assembly is the task of clustering the reads according to their origin, i.e. grouping together reads that sample the same haplotype. Specifically, reconstructing haplotypes of a *k*-ploid organism requires organizing the reads into *k* clusters—two clusters for diploids, three for triploids, etc. If sequencing is error-free, and the coverage/length of sequencing reads sufficiently high, clustering the reads (and, ultimately, reconstructing the haplotypes) would be rather straightforward (Bresler *et al.* 2013). However, sequencing is erroneous which makes haplotype assembly computationally challenging. The difficulty of haplotype assembly increases with the ploidy of the organism (Das and Vikalo 2015, Hashemi *et al.* 2018), as does the sequencing coverage required for accurate reconstruction. In particular, organizing the reads into clusters may be accomplished using a judiciously chosen measure of read similarity; however, limited read lengths and the presence of sequencing errors lead to ambiguous similarities and thus render the grouping of reads into clusters increasingly harder as the number of clusters (the ploidy) grows. Most of the existing haplotype assembly methods attempt to remove such ambiguities by altering or even discarding the data, leading to minimum SNP removal (Lancia *et al.* 2001), maximum fragments cut (Duitama *et al.* 2010), and minimum error correction (MEC) score (Lippert *et al.* 2002) optimization criteria. The majority of haplotype assembly methods developed in recent years are focused on optimizing the MEC score, i.e. determining the smallest possible number of nucleotides in sequencing reads that should be altered such that the resulting dataset is consistent with having originated from *k* haplotypes (*k* denotes the ploidy) (Chen *et al.* 2013, Kuleshov 2014, Pirola *et al.* 2015, Bonizzoni *et al.* 2016, Xie *et al.* 2016, Majidian *et al.* 2020, Moeinzadeh *et al.* 2020). Note that this formulation is known to be NP-hard (Schwartz 2010). One such algorithm, HapCUT (Bansal and Bafna 2008) constructs a read graph and seeks to find the max-cut that minimizes the MEC score. This was recently upgraded to HapCUT2 (Edge *et al.* 2017), which replaces the MEC score with haplotype likelihood; this formulation enables assembly from long reads. Haplotype assembly for polyploids (k>2) is more challenging than that for diploids (k=2) due to a much larger space of possible solutions. Techniques that can handle reconstruction of haplotypes for both diploid and polyploid genomes include HapTree (Berger *et al.* 2014), a Bayesian method that searches for the maximum likelihood estimate of the haplotypes by incrementally constructing a set of high-likelihood solutions, HapCompass (Aguiar and Istrail 2012), which seeks to find spanning trees to minimize read conflicts, H-PoP (Xie *et al.* 2016), a dynamic programming method that heuristically partitions reads to minimize differences between reads from the same haplotype and maximize the difference for reads from different haplotypes, and Ranbow (Moeinzadeh *et al.* 2020), a bottom-up approach to clustering reads and inferring haplotypes.

In this article, we present XHap, a novel method for haplotype assembly that relies on pairwise read correlations to cluster together sequencing reads, which originate from the same haplotype. The correlation between two reads, further discussed in Section 2, reflects the plausibility that the reads share their origin. XHap leverages transformers—powerful deep-learning models that rely on the so-called attention mechanism to discover long range dependencies in data (Vaswani *et al.* 2017)—to infer correlations between reads, including those do not overlap and may instead be separated by large genomic distances. In addition to haplotype assembly, the proposed framework for learning potentially non-measurable read correlations is suitable for other bioinformatics tasks made challenging by sequencing read length limitations, e.g. computational studies of viral quasispecies, bacterial communities, and intra-tumor heterogeneity.

## 2 Methods

### 2.1 Problem formulation

Sequencing platforms enable studies of genetic variations in a single individual organism by generating reads that essentially sample (with replacement) the organism’s chromosomes. Assuming that a reference genome is available, the relative positions of reads with respect to each other are readily determined by mapping them to the reference. Recall that the aim of single individual haplotyping is to reconstruct sequences of heterozygous alleles associated with each of the individual’s chromosomes. Since the frequency of point mutations on homologous chromosomes is relatively low (e.g. in the human genome, they are on the order of one polymorphism per thousand nucleotides) (Shastry 2007), large segments of reads do not cover any variant site; we discard those read segments along with the reads that cover only a single variant position and thus cannot help phase the haplotypes. The remaining read fragments are organized in an n×l matrix *S*, where *n* denotes the number of reads and *l* is the length of the haplotypes; each row of *S* corresponds to one of the reads, and the informative entries in a row are the heterozygous SNPs covered by the corresponding read. If a variant site is not covered by any read, the corresponding column of the constructed matrix will be empty; the matrix should then be split into two submatrices, one on each side of the column, corresponding to the haplotype blocks that will be reconstructed separately. Note that the data representation by means of a read fragment matrix accommodates reads of varied lengths. For convenience, we also represent the haplotypes by means of a k×l matrix *H*, where *k* denotes the number of haplotypes such that $H_{kl}$ is the lth allele of the kth haplotype. The goal of haplotype assembly can then be rephrased as follows: “Given the matrix of read fragments S, determine the matrix of haplotype sequences H.” XHap solves this problem by inferring pairwise read correlations and utilizing them to group together reads that “sample” the same haplotype. The correlation between two reads reflects the possibility that the reads originate from the same haplotype. For overlapping reads, a convenient proxy for this correlation is the relative difference between the number of alleles in which the reads agree and the number of those in which they differ; for a formal expression, please see (12) in Section 2.6. Intuitively, large positive values of read correlations (i.e. values close to +1) indicate that the reads likely originate from the same haplotype, while highly negative read correlations (values close to −1) suggest they come from different haplotypes. For non-overlapping reads, however, pairwise correlations cannot be measured—instead, XHap leverages the attention mechanism, commonly used in language processing, to discover correlations between even non-overlapping reads, to enable accurate haplotype reconstruction.

The majority of existing haplotype assembly methods aim to optimize the MEC score (Lippert *et al.* 2002), defined as the smallest number of nucleotides in the sequencing reads that would have to be changed so that the altered reads are consistent with the reconstructed haplotypes (i.e. each altered read is a subsequence of one of the haplotypes). Let HD(⋅,⋅) denote the Hamming distance between two overlapping sequences of nucleotides, defined as the sum of the Hamming distances between nucleotides in the corresponding positions of the two sequences; the Hamming distance between two nucleotides is zero if they are identical and one otherwise. If the reads are error-free, only the alleles at variant sites contribute to the computation of such Hamming distances. The MEC score of the reconstructed haplotype matrix $\hat{H}$ is defined as


(1) $$\text{MEC}(S,\hat{H})=\sum_{i=1}^{n}\underset{j=1,2,\ldots ,k}{\min}\text{HD}(S_{i},{\hat{H}}_{j}),$$

where $S_{i}$ denotes the ith read and ${\hat{H}}_{j}$ denotes the jth reconstructed haplotype. Intuitively, given $\hat{H}=({\hat{H}}_{1},{\hat{H}}_{2},\ldots ,{\hat{H}}_{k})$, the MEC score aggregates the distances from the reads to their respective closest reconstructed haplotypes. Haplotype assembly methods that pursue minimization of the MEC score essentially search for ${\hat{H}}_{j}$, j=1,2,…,k, that minimize (1). The MEC score is often used as a metric to characterize the accuracy of haplotype assembly methods, even if the design of those methods is not focused on minimizing the MEC score.

An alternative performance metric is the so-called correct phasing rate (CPR) (Hashemi *et al.* 2018). The CPR, also referred to as the reconstruction rate, is the average proportion of SNPs that are correctly reconstructed. Formally,


(2) $$\text{CPR}=1-\frac{1}{kl}\left(\underset{M}{\min}\sum_{i=1}^{k}\text{HD}(H_{i},M({\hat{H}}_{i}))\right),$$

where M is a one-to-one mapping from the reconstructed haplotypes to the true haplotypes. Note that CPR is a meaningful assessment of the accuracy of fully phased haplotypes. A related performance metric is the switch error rate (SWER) (Lin *et al.* 2004), defined for diploid assembly as the fraction of positions where the phase of the reconstructed haplotypes is erroneously switched. SWER is readily generalized to polyploids as the vector error rate (VER) (Berger *et al.* 2014, Schrinner *et al.* 2020). Note that among the aforementioned metrics, only the MEC can be computed in practical settings, where the ground truth is absent.

### 2.2 XHap: using a transformer to learn read correlations

XHap is an end-to-end pipeline that processes sequencing reads (short, long, or a combination thereof) and reconstructs the underlying haplotypes. As described in Section 2.1, sequencing reads are organized in an n×l SNP matrix *S*, where *n* denotes the number of reads and *l* is the length of the haplotypes (i.e. the number of heterozygous SNPs). This representation allows for reads of any length, so both short and long reads can seamlessly be incorporated in *S*. XHap takes *S* as the input and outputs the haplotypes as a k×l haplotype matrix, $\hat{H}$, where *k* denotes the number of haplotypes.

### 2.3 Convolutional encoder for embeddings

**Figure 1.**
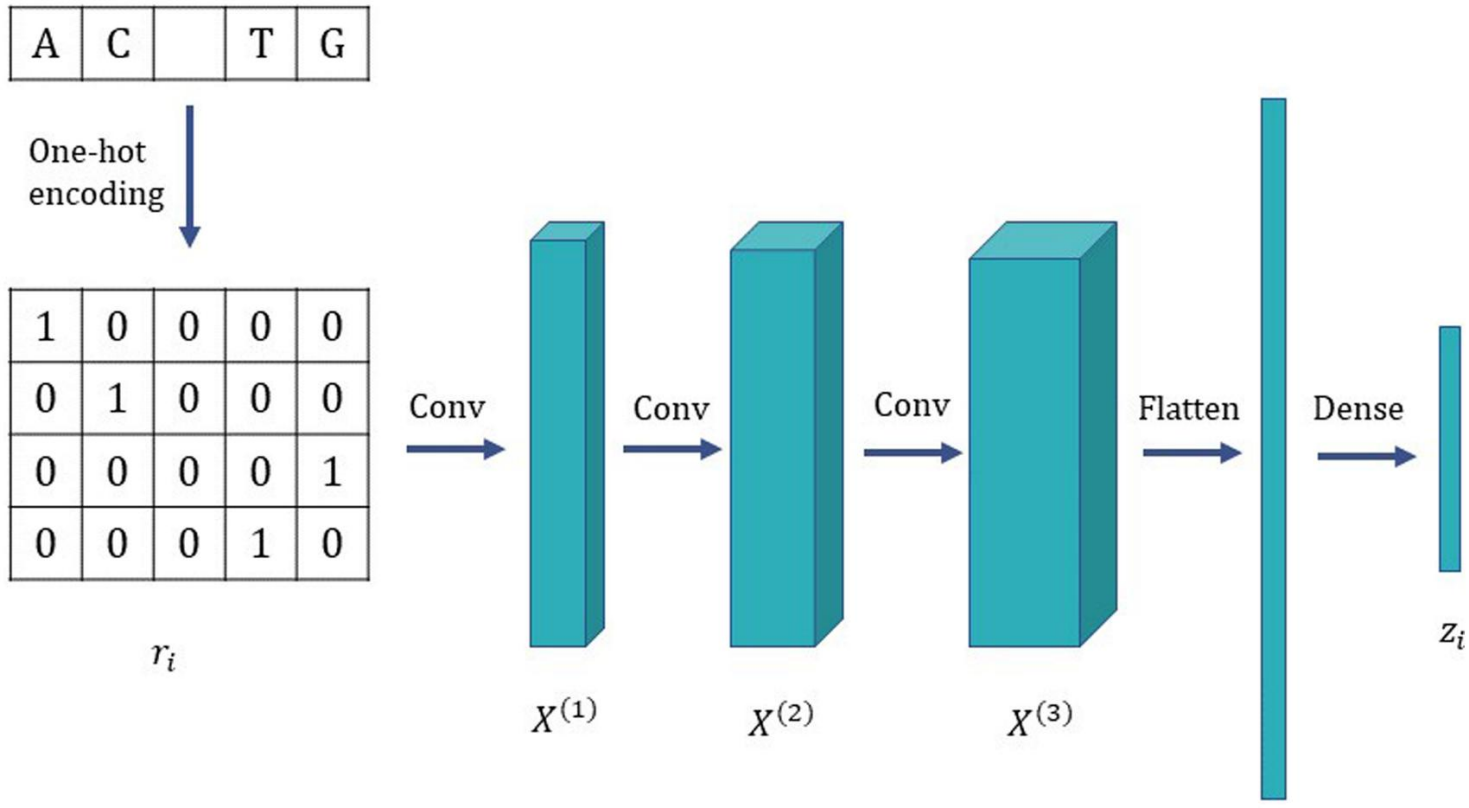
Architecture of the encoder for learning read embeddings. Here, $r_{i}$ denotes the one-hot encoded *i*-th row of the read matrix *S*. The convolutional encoder is trained to learn a $d_{r}$-dimensional latent representation of $r_{i}$.

The first stage of XHap is the convolutional encoder, adopted from CAECSeq (Ke and Vikalo 2020), to project the *l*-long-read representations in *S* to a lower dimension $d_{r}$. This is illustrated in Fig. 1. The reads in *S* are one-hot encoded by mapping each of the four possible nucleotides to vectors {(1,0,0,0),(0,1,0,0),(0,0,1,0), (0,0,0,1)}; genome positions not covered by any read are represented as (0,0,0,0). Such an encoding induces a symmetrical distance between the nucleotides. Then, the one-hot encoded reads are passed to a convolutional encoder to obtain read embeddings of the specified dimension $d_{r}$, as depicted in Fig. 1.

The convolutional encoder consists of three convolutional layers, followed by a dense layer. The operations of each of these layers can be formalized as


(3) $$\begin{array}{c} X^{\left(0\right)}=r_{i} \end{array},$$

(4) $$X^{\left(l\right)}=\sigma (X^{\left(l-1\right)}*W_{l-1}^{\mathrm{conv}}+B_{l-1}^{\mathrm{conv}}),l\in \{1,2,3\},$$

(5) $$\begin{array}{c} z_{i}=W_{1}^{\mathrm{dense}}\cdot \text{Flatten}(X^{\left(3\right)})+B_{1}^{\mathrm{dense}}, \end{array}$$

where $r_{i}$ denotes the one-hot encoding of the ith read and $z_{i}$ is the corresponding read embedding. $W_{l-1}^{\mathrm{conv}}$ and $B_{l-1}^{\mathrm{conv}}$ denote the weights and biases for the *l*-th convolutional layer, while $W_{1}^{\mathrm{dense}}$ and $B_{1}^{\mathrm{dense}}$ denote the weights and biases for the final dense layer in the encoder, respectively. Symbol “*” denotes the convolution operator and σ denotes the parametric rectified linear unit activation function (He *et al.* 2015). We set $d_{r}=128$, the kernel sizes of the layers are (4,5), (1,5), and (1,3), respectively, while the corresponding filter sizes are set to 32, 64, and 128. All strides are set to 1.

### 2.4 Transformer encoder for learning read correlations

The next block in the XHap computational pipeline is the transformer encoder, which learns the correlations between the reads by utilizing the encoder layers in Vaswani *et al.* (2017) as a building block. Specifically, XHap deploys three such layers, followed by a dense, layer which outputs read correlations (see Fig. 3). The learned read embeddings $z_{i}$ are first stacked to form the read embedding matrix *Z*. Since the transformer requires a 3D tensor as input, *Z* is further padded with a dummy dimension to form $E^{\left(0\right)}$.

#### 2.4.1 A Transformer encoder layer

**Figure 2.**
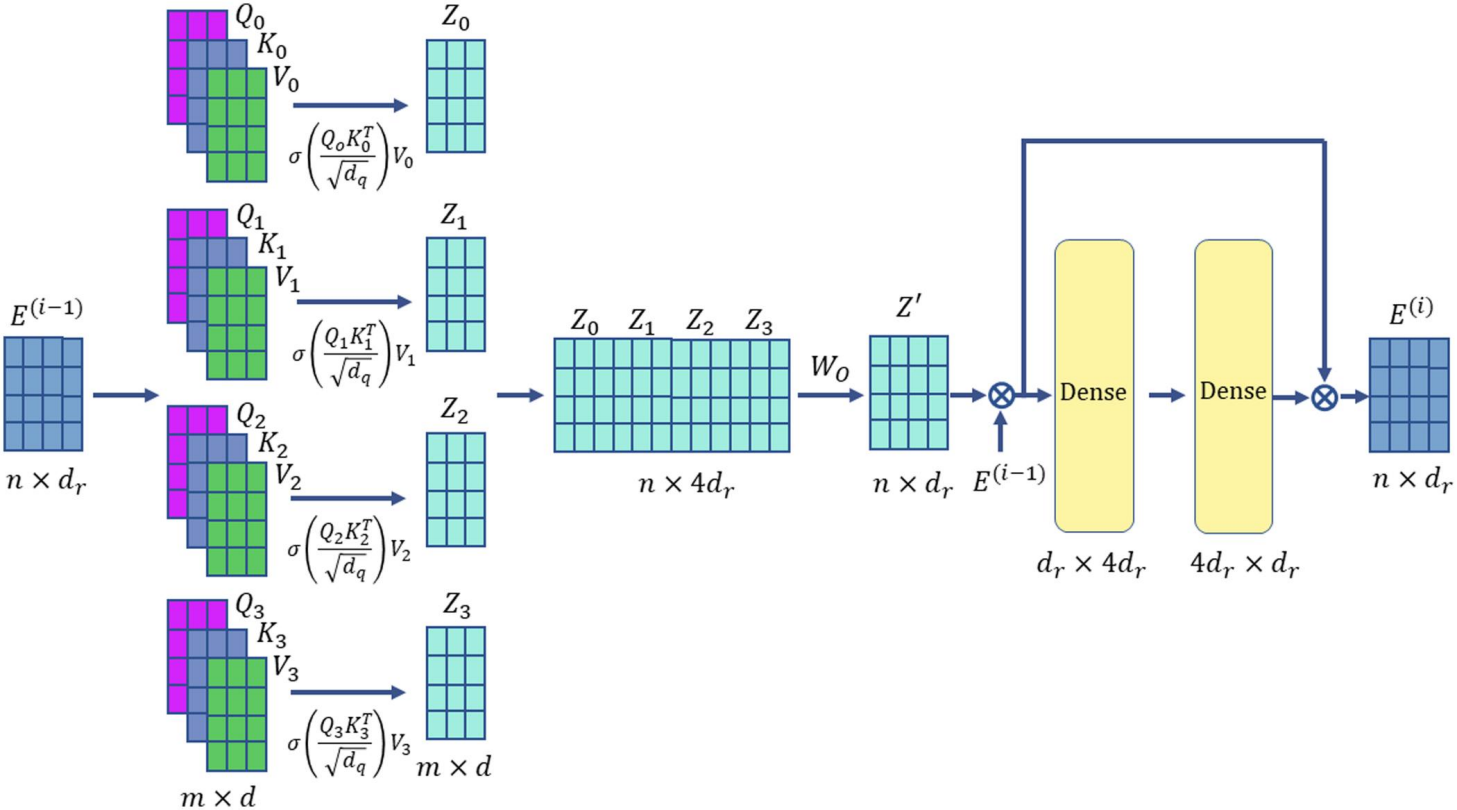
An illustration of the transformer encoder layer. The adder denotes the “add and layer-normalize” operation.

We adopt the concept of multi-headed self-attention (Vaswani *et al.* 2017) to learn the relationships between reads. In the context of haplotype assembly, “self-attention” refers to the notion that a read is associated with other reads to varying degrees. The encoder quantifies self-attention by computing query ($Q_{i})$, key ($K_{i}$), and value ($V_{i}$) matrices, and subsequently deriving the self-attention matrices $Z_{i}$. Each layer of the encoder employs multiple “attention heads” of this form. The self-attention matrices are concatenated and condensed to form the output of the multi-headed self-attention, $Z^{\prime }$. At this stage, the input matrix is added to $Z^{\prime }$ via a residual connection and layer-normalized before being passed through dense layers. Finally, the output of the encoder layer is obtained by adding the input and output of the dense layers and layer-normalizing the result. As shown in Fig. 2, we use four attention heads in each encoder layer. The use of multiple attention heads improves the likelihood that the learned self-attention weights are meaningful. A likely scenario is that, for a given read, one self-attention head concentrates most of its weight on the read itself but the other attention heads have weights spread across other reads. As previously mentioned, the transformer encoder consists of three such layers.

#### 2.4.2 Architecture of the transformer encoder

**Figure 3.**
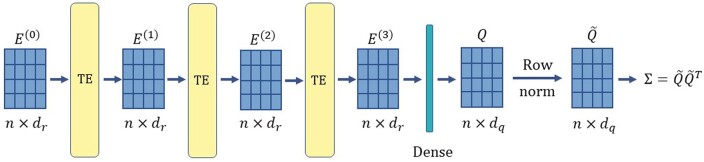
Architecture of the encoder used to learn read embeddings. TE denotes the standard transformer encoder layers. The described encoder takes the read embedding matrix $Z\in R^{n\times d_{r}}$ and returns a read-correlation matrix $\Sigma \in R^{n\times n}$.

Recall that XHap aims to learn pairwise read correlations. Hence, the output of the last encoder layer is piped to a dense layer containing $d_{q}$ neurons, whose output is a matrix $Q\in R^{n\times d_{q}}$. We normalize each row of *Q* to form $\tilde{Q}$ so that the learned read-correlation matrix $\Sigma =\tilde{Q}{\tilde{Q}}^{T}$ has one as its diagonal elements. Such a construction ensures that Σ is positive semi-definite and, consequently, a valid kernel. Denoting the transformer encoder layers as TE(⋅), we can formalize the operations of the transformer encoder as


(6) $$\begin{array}{c} E^{\left(0\right)}=\text{Expand}(Z) \end{array},$$

(7) $$E^{\left(l\right)}=\text{TE}(E^{\left(l-1\right)}),\;\;l\in \{1,2,3\},$$

(8) $$\begin{array}{c} Q=W_{2}^{\mathrm{dense}}\cdot E^{\left(3\right)}+B_{2}^{\mathrm{dense}} \end{array},$$

(9) $$\tilde{Q}=\text{RowNormalize}(Q),$$

(10) $$\begin{array}{c} \Sigma =\tilde{Q}{\tilde{Q}}^{T}, \end{array}$$

where *Z* is an $n\times d_{r}$ matrix with its ith row set to $z_{i}$. Expand() denotes the operation of adding a dummy dimension, i.e. setting the batch size to one. $W_{2}^{\mathrm{dense}}$ and $B_{2}^{\mathrm{dense}}$ denote the weights and biases of the dense layer in Fig. 3, respectively. The dimension of *Q* is set to be $n\times d_{q}$, where $d_{q}=d_{r}/2=64$.

### 2.5 Haplotype assembly enabled by read clustering

We formulate the problem of attributing reads to their respective haplotypes as a clustering task. To this end, we rely on kernel *k*-means (Zhang and Rudnicky 2002, Dhillon *et al.* 2004, Welling 2013), using Σ as the kernel to cluster the reads; each resulting cluster corresponds to a distinct haplotype. Kernel *k*-means clustering expands upon conventional *k*-means by grouping input data points in an implicit higher-dimensional feature space. The haplotype corresponding to a cluster is formed as the consensus sequence across reads attributed to that cluster.

### 2.6 Putting the pieces together: optimizing XHap

Recall that the algorithmic goal of XHap is to associate reads with the underlying haplotypes in an “unsupervised” manner, i.e. determine the read attributions in the absence of ground truth. We accomplish this by training the neural network in an alternating fashion. Specifically, each training epoch consists of the following two steps:

1. using the read attributions from the last epoch, train the convolutional encoder and the transformer encoder to learn the read-correlation matrix Σ; and
2. relying on the learned read-correlation matrix, organize the reads using kernel *k*-means into *k* clusters to obtain read attributions.

#### 2.6.1 Contrastive loss

For training the convolutional and transformer encoder, XHap relies on the contrastive loss (Hadsell *et al.* 2006). In particular, for any pair of reads *i* and *j*, we introduce a variable $p_{ij}$ and set $p_{ij}=1$ if the two reads are attributed to the same haplotype; then, the contrastive loss is defined as


(11) $$L_{c}=\sum_{i,j}\left[p_{ij}{\left(1-\Sigma_{ij}\right)}^{2}+(1-p_{ij}){\left(1+\Sigma_{ij}\right)}^{2}\right],$$

where $\Sigma_{ij}$ denotes the (i,j) element of Σ. Intuitively, contrastive loss promotes larger $\Sigma_{ij}$ for reads *i* and *j* originating from the same haplotype, and pushes the others to −1. As the contrastive loss is evaluated over all read pairs, this intuition extends to non-overlapping read pairs; as long as there are a set of overlapping reads spanning the gap between the non-overlapping read pair, the contrastive loss enables the propagation of the polarity of the read correlations to $\Sigma_{ij}$ for the non-overlapping read pair. Note that at any time step, $p_{ij}$ is computed from the cluster attributions obtained in the previous epoch.

#### 2.6.2 Regularizers

We incorporate regularizers in the loss function to enforce consistency of Σ with the data, and to promote finding Σ reflective of the setting where each read exhibits strong positive or negative correlation with only a subset of the reads while experiencing relatively weaker correlation with the remaining reads. For the former, we first pre-compute the values of correlations for each pair of overlapping reads *i* and *j* in the following way (Das and Vikalo 2015): if $k_{\mathrm{sim}}$ is the number of overlapping positions in which the two reads agree, and $k_{\mathrm{dissim}}$ is the number of overlapping positions in which they differ, the correlation between reads *i* and *j* is computed as


(12) $$C_{ij}=\frac{k_{\mathrm{sim}}-k_{\mathrm{dissim}}}{k_{\mathrm{sim}}+k_{\mathrm{dissim}}}.$$

For non-overlapping reads, $C_{ij}$ is trivially zero; for overlapping reads, correlations $C=[C_{ij}]$ exhibit the trends that we expect to observe in Σ, i.e. $C_{ij}$ takes on positive values when reads are similar at the overlapping positions and negative when they differ at those positions. Then, we adopt the regularizer


(13) $$L_{r}=||\Omega_{C}(\Sigma -C)||,$$

where $\Omega_{C}(\cdot )$ denotes a mask selecting only the indices where $C_{ij}\neq 0$. Such a regularization promotes finding Σ that agrees with *C* on the entries (i,j) for each pair of overlapping reads *i* and *j* while allowing XHap to discover hitherto non-measurable read correlations, i.e. correlations between non-overlapping reads.

Moreover, we introduce a regularizer promoting sparsity in the learned Σ; this incentivizes solutions where each read is strongly correlated to only a small subset of other reads. Such a restriction confers two key benefits: (i) it effectively reduces the parameter space that has to be explored when training the full network, and (ii) it reduces the difficulty of the read clustering step. The sparsity regularization is facilitated using


(14) $$L_{s}=\sum_{i}\sum_{j\neq i}|\Sigma_{ij}|.$$

Essentially, (14) promotes Σ with small off-diagonal elements. Finally, the overall loss function is formed as $L=L_{c}+\lambda_{r}L_{r}+\lambda_{s}L_{s}$. A formalization of the proposed algorithm, XHap, can be found in Supplementary Section A.

#### 2.6.3 Hyperparameter and model selection

Preliminary simulated data at 30× coverage was generated as outlined in Section 3.1. This was then used to train XHap, and the hyperparameters that yielded the lowest MEC were selected for all subsequent experiments. For each dataset, XHap is randomly initialized five times and trained; the model that yields the lowest MEC was selected. For all the reported simulation results, $\lambda_{r}=100$ and $\lambda_{s}=10$. The batch size is set to $\left\lceil \frac{n}{5}\right\rceil$ and optimized using the Adam optimizer (Kingma and Ba 2014) with a learning rate of $10^{-5}$ over 2000 epochs. These hyperparameters remained fixed in all subsequent experiments, both on short and long reads. The experiments on semi-experimental and real data were conducted on an AMD Vega 20 GPU on a server with 96 1.50 MHz AMD EPYC 7642 processors.

## 3 Results

### 3.1 Haplotype assembly with XHap from short (Illumina) sequencing reads

We compare the performance of XHap on haplotype assembly from short sequencing reads to those of state-of-the-art methods including CAECSeq (Ke and Vikalo 2020), H-PoP (Xie *et al.* 2016), HapTree (Berger *et al.* 2014), HapCUT2 (Edge *et al.* 2017), and Ranbow (Moeinzadeh *et al.* 2020). Adopting the pipeline outlined in Motazedi *et al.* (2018), we select at random a 10 kb region of the *Solanum tuberosum* Chromosome 5 as the reference. Then, the log-normal model from Haplogenerator (Motazedi *et al.* 2018) is used to introduce independent mutations, creating *k* sequences, where *k* denotes the ploidy (k=2 for diploid, k=3 for triploid, etc.). The mean log-distance between successive mutations and the corresponding standard deviation are set to 3.03 and 1.293, respectively; this corresponds to a mean SNP distance of 50 bp (Sevestre *et al.* 2020) and yields haplotypes of approximate length 200 variants. This particular choice of the SNP distance matches the expected distance between SNPs in the *S.tuberosum* genome (Uitdewilligen *et al.* 2013). Next, ART (Huang *et al.* 2012) is used to generate paired-end MiSeq reads of length 2×250 bp with mean insert length 550 bp and standard deviation 10 bp. The reads are mapped to the reference using BWA-MEM (Li and Durbin 2009). SNP positions are identified as the sites where the frequency of alternative allele(s), i.e. the variant allele frequency (VAF), exceeds a predefined threshold. In the conducted experiments, this threshold is set to 0.2. Sequencing coverage is varied from 10× to 30× in steps of 10×, resulting in read counts ranging from around 200 to 600. All the randomized algorithms were run with five random initializations for each coverage setting, and the haplotypes for the initialization that yields the lowest MEC score are recorded. For each sequencing coverage, the reported results include the mean and standard deviation of all the metrics across five simulated datasets. Some of the existing methods for haplotype reconstruction do not phase complete haplotypes, so the true CPR and SWER/VER cannot be computed. In lieu of that, the tables report the average CPR and SWER over the haplotype blocks. These entries are marked with *. The best scores on each metric have also been highlighted in bold.

#### 3.1.1 Diploid (k=2) assembly

**Figure 4.**
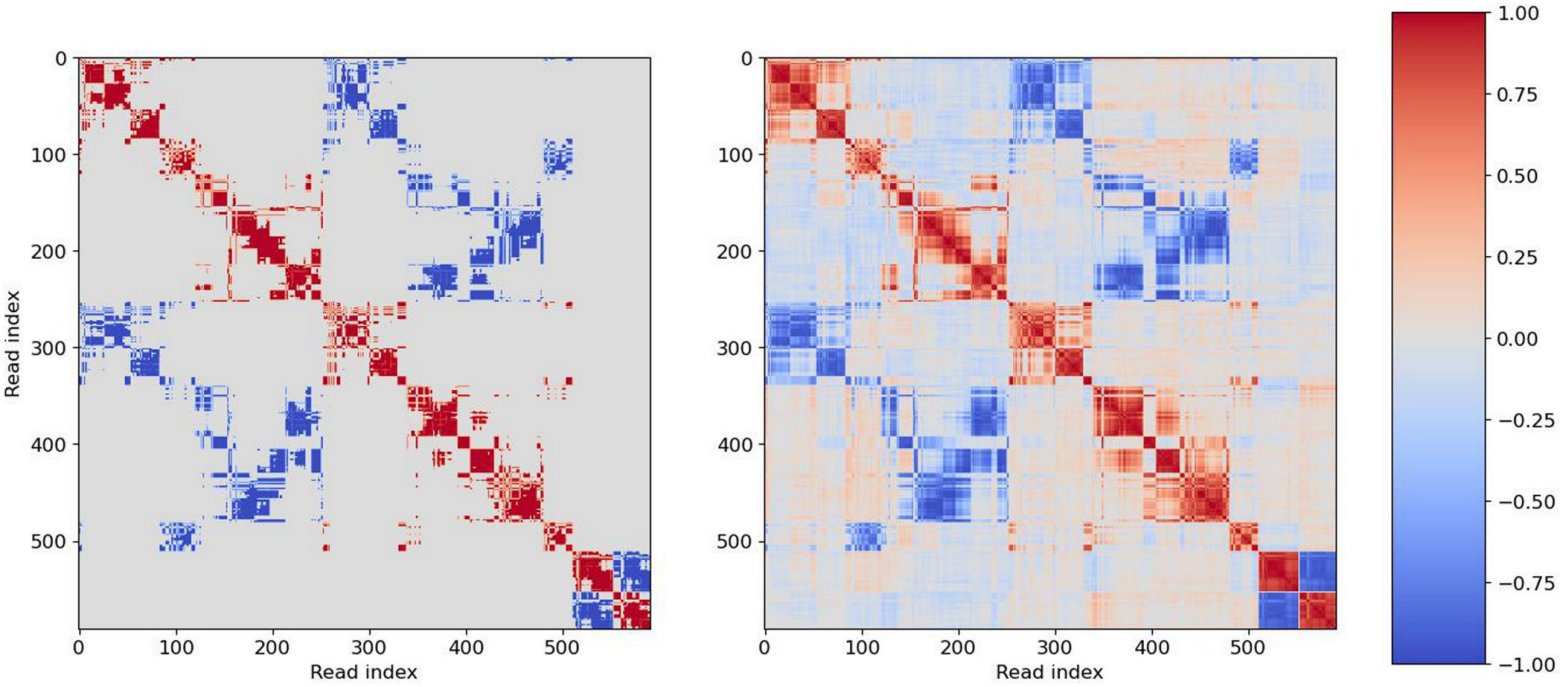
The heat map of read correlations; the axes show the indices of reads ordered via single-linkage clustering based on the measured correlations. The plot on the left shows the correlations that can be computed from pairwise read overlaps ($C_{ij}$). The plot on the right shows the correlations inferred by XHap ($\Sigma_{ij}$); XHap “inpaints” the map on the right starting from the one on the left by propagating information from the overlapping reads to the non-overlapping ones and estimating correlations between pairs of reads separated by potentially large genomic distances. In the displayed example, XHap is able to infer correlations >0.1 for reads that are as far as 8500 bp apart and correlations >0.25 for reads up to 7500 bp apart.

As can be seen from Table 1, XHap successfully assembles complete haplotypes, significantly outperforming competing methods in terms of the considered metrics. Notably, at higher coverages XHap attains near-perfect reconstruction, achieving a CPR ≈1. In contrast, most of the other considered algorithms can only assemble fragments of the haplotypes, returning multiple blocks; even on these smaller blocks, XHap achieves higher reconstruction accuracy. XHap accomplishes this by estimating non-measurable read correlations, i.e. by inferring correlations between non-overlapping reads. Figure 4 illustrates measured (left) and transformer-inferred (right) pairwise read correlations. For the sake of visualization, the reads with higher measured correlations are indexed close to each other. XHap can be thought of as “inpainting” the plot on the right (replacing gray in the left plot by red/blue in the right plot), facilitating propagation of the information from overlapping to non-overlapping reads. Notably, inpainting of the off-diagonal blocks in the plot on the right of Fig. 4 is due to XHap estimating correlation between pairs of reads that do not overlap and may in fact be separated by large genomic distances.

**Table 1.** Performance of XHap, CAECSeq, HapTree, H-PoP, and HapCUT2 on semi-experimental diploid data (short reads) as the sequencing coverage varies.

	Coverage	MEC	CPR	SWER	Blocks
XHap	10	**35 ± 9.36**	**0.900 ± 0.146**	**0.006 ± 0.006**	1
	20	**18 ± 7.18**	**0.995 ± 0.005**	**0.001 ± 0.003**	1
	30	**20 ± 6.8**	**0.999 ± 0.001**	**0**	1
CAECSeq	10	58 ± 14.8	0.614 ± 0.032	0.008 ± 0.006	1
	20	29 ± 7.03	0.717 ± 0.149	0.006 ± 0.005	1
	30	46 ± 32.5	0.729 ± 1.109	0.005 ± 0.002	1
H-PoP	10	67 ± 18.4	0.922 ± 0.100*	0.004 ± 0.007*	4.60 ± 1.74
	20	60 ± 27.8	0.988 ± 0.005*	$0^{*}$	3.20 ± 1.17
	30	136 ± 54.2	0.983 ± 0.007*	0.000 ± 0.001*	6.00 ± 1.10
HapCUT2	10	96 ± 31.7	0.908 ± 0.022*	0.024 ± 0.015*	6.80 ± 3.25
	20	63 ± 29.8	0.981 ± 0.014*	0.005 ± 0.011*	1.80 ± 1.17
	30	137 ± 54.2	0.980 ± 0.007*	0.002 ± 0.002*	2.40 ± 1.02
HapTree	10	90 ± 29.5	0.773 ± 0.189*	0.039 ± 0.015*	1.80 ± 0.75
	20	61 ± 28.6	0.982 ± 0.011*	0.005 ± 0.011*	1.20 ± 0.40
	30	137 ± 53.4	0.981 ± 0.006*	0.003 ± 0.003*	2.20 ± 0.98

#### 3.1.2 Triploid (k=3) assembly

We now turn our attention to the more challenging problem of reconstructing polyploid haplotypes. Table 2 reports the performance of XHap and the competing methods on triploid (k=3) semi-experimental data. Note that HapCUT2 and HapTree, whose performance in application to assembly of diploid haplotypes is reported in Table 1, are omitted here as the current releases of these software support only diploid reconstruction. At higher coverage, XHap outperforms existing algorithms in terms of the MEC score. As in the diploid case, H-PoP is unable to phase the entire haplotype and instead returns multiple blocks. At high coverage, XHap’s VER, evaluated over the fully phased haplotype, achieves VER comparable to the VER attained by H-PoP averaged across its blocks.

**Table 2.** Performance of XHap, CAECSeq, H-PoP, and Ranbow on semi-experimental triploid data (short reads) as the sequencing coverage varies.^a^

	Coverage	MEC	CPR	VER	Blocks
XHap	10	71 ± 8.89	0.701 ± 0.049	0.040 ± 0.016	1
	20	**55 ± 15.7**	**0.713 ± 0.039**	**0.013 ± 0.002**	1
	30	**64 ± 17.6**	**0.826 ± 0.077**	**0.009 ± 0.005**	1
CAECSeq	10	82 ± 18.3	0.715 ± 0.051	0.060 ± 0.015	1
	20	90 ± 29.5	0.665 ± 0.053	0.026 ± 0.007	1
	30	118 ± 51.0	0.736 ± 0.06	0.024 ± 0.012	1
H-PoP	10	**67 ± 24.4**	0.826 ± 0.103*	0.020 ± 0.008*	3.20 ± 1.47
	20	73 ± 30.8	0.913 ± 0.065*	0.005 ± 0.005*	3.40 ± 0.80
	30	114 ± 41.4	0.882 ± 0.103*	0.007 ± 0.007*	2.60 ± 1.74
Ranbow	10	306 ± 65			
	20	575 ± 170			
	30	849 ± 106			

a Ranbow reports an incorrect number of haplotypes, which is why several performance metrics for this method could not be computed.

### 3.2 Haplotype assembly from long reads: robustness to sequencing errors

Next, we consider the problem of haplotype assembly from long sequencing reads such as those generated by PacBio and ONT devices. These reads span far greater genomic distances than short reads but do so at the expense of accuracy; in particular, the error rates of long reads are an order of magnitude higher than the error rates of short reads (often exceeding 5%). The high rate of sequencing errors exacerbates the difficulty of the haplotype assembly problem. PacBio read synthesis is emulated here using the PBSIM2 simulator (Ono *et al.* 2021). To this end, we first select at random a 100 kb region of the human GrCh38 genome, and proceed to generate the haplotypes similarly to the process outlined in Section 3.1. The mean and standard deviation of the log-normal model was set to 6.07 and 1.293, respectively, which translates to a mean SNP distance of 1000 bp and an average haplotype length of 100. With the default settings of PBSIM2, we generate reads of an average length of 9000 bp. The reads are subsequently aligned to the reference using BWA-MEM (Li and Durbin 2009); the sites with VAFs exceeding 0.2 are called as SNPs. The sequencing coverage is varied from 80× to 100× in steps of 10×; this corresponds to a read count ranging from 880 to 1100. The coverage was selected to ensure accurate variant calling; at low coverage, the high read error rate makes variant calling challenging. All the randomized algorithms were run with five random initializations for each coverage setting, and the haplotypes for the initialization that yields the lowest MEC score are recorded. For each sequencing coverage, the reported results consist of the mean and standard deviation of all the metrics for the five simulated datasets.

#### 3.2.1 Diploid (k=2) assembly

As can be seen in Table 3, XHap outperforms other algorithms in terms of all the metrics and in all settings except at the lowest coverage where CAECSeq has an edge. The CPR and SWER achieved by XHap are better than the average CPR and SWER of the haplotype fragments returned by HapTree, H-PoP, and HapCUT2, yet by phasing complete haplotypes, XHap solves a more difficult problem.

**Table 3.** Performance of XHap, CAECSeq, HapTree, H-PoP, and HapCUT2 on semi-experimental diploid data (PacBio long reads) as the sequencing coverage varies.

	Coverage	MEC	CPR	SWER	Blocks
XHap	80	449 ± 151.6	0.834 ± 0.033	**0.004 ± 0.006**	1
	90	**553 ± 68.3**	**0.873 ± 0.066**	0.004 ± 0.003	1
	100	**587 ± 86.5**	**0.882 ± 0.036**	**0**	1
CAECSeq	80	**447 ± 152.6**	**0.847 ± 0.053**	**0.004 ± 0.004**	1
	90	575 ± 72.7	0.824 ± 0.137	0.002 ± 0.003	1
	100	623 ± 111.2	0.778 ± 0.127	0.003 ± 0.003	1
H-PoP	80	573 ± 101.8	0.703 ± 0.061*	0.009 ± 0.007*	21.40 ± 6.18
	90	765 ± 80.8	0.769 ± 0.106*	0.003 ± 0.002*	15.8 ± 8.91
	100	755 ± 149.7	0.795 ± 0.068*	0.004 ± 0.006*	12.80 ± 9.24
HapCUT2	80	526 ± 104.0	0.840 ± 0.040*	0.003 ± 0.003*	4.80 ± 1.94
	90	724 ± 94.0	0.845 ± 0.058*	0.006 ± 0.005*	4.20 ± 2.99
	100	726 ± 140.9	0.846 ± 0.031*	0.009 ± 0.009*	3.80 ± 1.47
HapTree	80	515 ± 107.9	0.852 ± 0.034*	0.004 ± 0.004*	1.20 ± 0.40
	90	717 ± 98.3	0.854 ± 0.051	0.008 ± 0.004	1
	100	719 ± 139.8	0.856 ± 0.032	0.012 ± 0.009	1

#### 3.2.2 Triploid (k=3) data

Table 4 compares the performance of XHap and the competing algorithms on haplotype assembly of triploids from long reads. In all settings, XHap achieves the best MEC scores. A closer inspection of CPR and VER suggests that at low coverage, XHap may switch haplotype blocks—such errors are penalized harshly in CPR but not in VER. Meanwhile, H-PoP struggles to assemble polyploid haplotypes from long reads, as indicated by the significantly poorer MEC scores and CPR. In general, the two deep-learning algorithms, XHap and CAECSeq, achieve the lowest MEC scores across the board. CAECSeq narrowly outperforms XHap in terms of CPR while both exhibit superior performance compared to H-PoP and Ranbow.

**Table 4.** Performance of XHap, CAECSeq, and H-PoP on semi-experimental triploid data (PacBio long reads) as the sequencing coverage varies.^a^

	Coverage	MEC	CPR	VER	Blocks
XHap	80	**117 ± 32.5**	**0.731 ± 0.056**	**0.015 ± 0.010**	1
	90	**102 ± 29.2**	0.751 ± 0.073	**0.025 ± 0.014**	1
	100	**157 ± 30.5**	0.698 ± 0.020	**0.011 ± 0.007**	1
CAECSeq	80	159 ± 55.5	0.726 ± 0.032	0.034 ± 0.007	1
	90	225 ± 139	**0.758 ± 0.034**	0.047 ± 0.029	1
	100	260 ± 139	**0.773 ± 0.053**	0.042 ± 0.056	1
H-PoP	80	239 ± 89.9	0.512 ± 0.080*	0.008 ± 0.004*	17.6 ± 9.11
	90	241 ± 73.4	0.530 ± 0.150*	0.031 ± 0.017*	13.2 ± 5.34
	100	275 ± 42.1	0.500 ± 0.080*	0.014 ± 0.009*	16.0 ± 5.48

a Since Ranbow reconstructed only very small segments of haplotypes, its results are omitted.

### 3.3 Performance on experimental *S.tuberosum* data

Performance of XHap is further tested on real *S.tuberosum* data (k=4) available under NCBI accession SRR6173308. This dataset contains paired-end Illumina HiSeq 2000 reads (2×100bp in length) obtained by sequencing *S.tuberosum* Chromosome 5  (Xu *et al*. 2011). Five regions, each 10 kb long, are selected at random from the reference genome. The reads are mapped using BWA-MEM (Li and Durbin 2009) to these references, followed by SNP calling with a threshold on VAF set to 0.1. The number of reads and SNPs in each region are shown in Supplementary Table S1. In the absence of ground truth, one can characterize the quality of haplotype reconstruction via the considered algorithms only by means of the MEC scores. As can be seen in Table 5, XHap outperforms all other methods on four out of five considered regions.

**Table 5.** The MEC scores of XHap, CAECSeq, H-PoP, and Ranbow on real *S.tuberosum* data.

Region	1	2	3	4	5
XHap	967	**54**	**379**	**261**	**16**
CAECSeq	**922**	89	436	282	73
H-PoP	1191	352	486	412	222
Ranbow	1159	207	656	382	204

### 3.4 Performance on experimental human data

Further validation of XHap was performed on the NA12878 dataset provided by the Genome in a Bottle consortium (Zook *et al.* 2016). This dataset contains aligned PacBio SMRT whole-genome read data at a coverage of 44× for a human subject. We followed the benchmarking practices outlined in Wagner *et al.* (2022) and selected only the high-confidence SNP calls for our analysis. The included VCF file contains phased genotype calls, which are then used to form the ground truth haplotypes. For our input, we first segregated the reads by chromosome and then selected only the reads covering the high-confidence regions. This yields 161 072 reads covering 28 642 SNPs for Chromosome 22. Owing to the large size of the read fragment matrix, we run XHap to reconstruct overlapping haplotype blocks and phase them together to obtain the complete reconstructed haplotypes. Specifically, XHap reconstructs blocks of length 250 SNPs with successive blocks overlapping by 50 SNPs. This results in a model comprising 8.63 million parameters. Reads within each block covering <2 SNPs were discarded as they are uninformative for haplotype reconstruction within the given block. Successive haplotype blocks were then phased together by finding the best match between the previously recovered haplotypes over the last 50 SNPs and the newly reconstructed haplotypes over the first 50 SNPs. Observe that each haplotype block can be reconstructed independently; we leverage this by reconstructing four blocks in parallel, each on a different GPU.

Results for assembly on Chromosome 22 are shown in Table 6. Results for HapTree could not be obtained as the software resulted in an error when run on this data. Here too, we observe that XHap reconstructs haplotypes with the lowest MEC score amongst all the algorithms. Moreover, HapCUT2 and H-PoP fragment the haplotype into numerous blocks (nearly 300) while XHap reconstructs complete haplotypes with only a marginal drop in the SWER.

**Table 6.** Performance of XHap, CAECSeq, H-PoP, and HapCUT2 on PacBio SMRT reads for Chromosome 22 of the NA12878 genome.

	MEC	SWER	Blocks	CPU time (min)
XHap	**109 902**	0.003	1	3300
CAECSeq	148 798	0.017	1	214 690
H-PoP	110 060	0.001*	285	0.13
HapCUT2	113 676	0.002*	298	0.34

## 4 Discussion

The proposed algorithm, XHap, reconstructs haplotypes from sequencing reads by inferring read correlations through the use of transformers. To facilitate this inference, reads are first projected onto a lower-dimensional space using a convolutional encoder. Subsequently, read correlations and haplotype memberships of the reads are learned by alternating between the following two steps: (i) reads from the same haplotype are clustered together via kernel *k*-means using read correlations as similarity measures, and (ii) read memberships are used to refine the correlations learned by the transformer. The learned read correlations are particularly beneficial when performing assembly from short reads—in this setting, only a relatively small fraction of reads overlap. Therefore, learning correlations between non-overlapping reads leads to superior performance of XHap over existing algorithms for haplotype assembly from short reads; at high coverage, in particular, XHap achieves near-perfect haplotype reconstruction. XHap also generally outperforms competing methods in haplotype assembly tasks where the data comes from long-read sequencing platforms. Moreover, we observe that as long as there is a read bridging each variant position, XHap returns completely phased haplotypes; this stands in contrast to competing methods, which are often unable to fully phase haplotypes and instead return them fragmented into blocks. While XHap considers only SNPs when reconstructing haplotypes, an additional pipeline to detect insertions and deletions that may exist in one of the reconstructed sequences has been provided in Supplementary Section C, along with preliminary results validating performance of the pipeline.

## 5 Conclusion

XHap leverages transformers, a powerful deep-learning architecture that originated in the field of natural language processing, to learn read dependencies en route to assembling haplotypes. The experiments on realistic simulated as well as experimental data demonstrate XHap’s ability to discover correlations between reads separated by large genomic distances, ultimately leading to significant improvement in the quality of the reconstructed haplotypes. We anticipate that learning non-measurable read correlations may enable algorithmic advancements in other tasks that are rendered challenging due to limitations on sequencing read lengths, including problems of reconstructing viral quasispecies, analyzing bacterial communities, and painting the genomic landscape of cancer cells.

## Supplementary Material

vbad169_Supplementary_Data

## Data Availability

The code implementing XHap and scripts for data generation and replicating the included experiments are publicly available at https://github.com/shoryaconsul/XHap. The *Solanum tuberosum* data used in this manuscript can be downloaded from https://www.ncbi.nlm.nih.gov/sra/SRR6173308. The GIAB NA12878 dataset can be found at https://ftp-trace.ncbi.nlm.nih.gov/ReferenceSamples/giab/data/NA12878/NA12878_PacBio_MtSinai/.
