# Supplementary material for: XHap: haplotype assembly using long-distance read correlations learned by transformers
Source: Bioinform Adv. 2023 Nov 23;3(1):vbad169. doi: 10.1093/bioadv/vbad169 (PMC10713121; doi:10.1093/bioadv/vbad169)
Supplement: vbad169_Supplementary_Data [file vbad169_supplementary_data.pdf]

Supplementary Information

A. Algorithm

The proposed XHap algorithm is formalized as Algorithm 1.

Algorithm 1 XHap: Haplotype assembly using correlations learned by transformers

Input: Read fragment matrix $S \in \{A, C, G, T\}^{n \times l}$, the number of haplotypes k, max number of epochs T, regularization constants λ_r and λ_s

Output: Matrix of haplotype sequences $H \in \{A, C, G, T\}^{k \times l}$

1: Obtain expanded read tensor $R \in \{0, 1\}^{n \times l \times 4}$ by one-hot encoding bases in the reads

2: Compute $C = [C_{ij}]$ as

$$C_{ij} = \frac{k_{sim} - k_{dissim}}{k_{sim} + k_{dissim}},$$

where k_{sim} and k_{dissim} denote the number of similar and dissimilar overlapping positions between reads i and j, respectively.

3: Randomly assign each read to one of k haplotypes.

4: **for** $i = 1 \leftarrow 1, T$ **do**

5: Train the encoder and transformer encoder using the loss function $L = L_c + \lambda_r L_r + \lambda_s L_s$ given the current read attributions.

6: Cluster reads into k groups using Σ as the kernel for kernel k-means.

7: Determine consensus haplotypes for each group to form the haplotype matrix $H^{(i)}$.

8: Compute MEC for $H^{(i)}$.

9: **end for**

10: $H \leftarrow \arg\min_{H^{(i)}} \text{MEC}\left(S, H^{(i)}\right)$

B. Details on the selected regions of real **Solanum tuberosum** data

Table S1. The number of reads and SNPs for the selected regions of *Solanum tuberosum* genome.

Region	1	2	3	4	5
Number of SNPs	217	182	284	215	132
Number of reads	586	73	165	252	79

C. Indel detection

In addition to single nucleotide polymorphisms, the genome of an organism may experience insertions and deletions. In this section, we outline approaches to detecting indels from short or long sequencing reads, focusing on indels of intermediate size (on the order of 100bp); such indels are detected and reconstructed directly from read alignments. Longer indels can be detected through the use of existing tools, such as HaplotypeCaller (Poplin et al., 2017), which reconstruct inserted regions through localized de-novo assembly.

Our indel detection pipeline operates on the set of reads aligned to the reference genome using BWA-MEM. At a high level, the pipeline consists of two steps:

1. Infer the origin of aligned reads.
2. Determine the length of indel and, in case of insertion, recover the inserted sequence for each haplotype.

C.0.1. Inferring read origin.

In order to infer the origin of a read, we compute the Hamming distance of the aligned parts of the read to each of the haplotypes. The haplotype with the smallest distance to a read is declared to be the origin of the read.

C.0.2. Indel detection from short reads.

When reads arising from a region containing the indel are aligned to the reference, the aligner outputs split alignments, as shown in Fig. 1.

Deletion:
In the event of deletion between reference positions x_1 and x_2 (see Fig. 1a), the aligner 'splits' the read into two smaller read segments, r and r', to improve the quality of the alignment. In particular, read segment r is aligned such that it ends at position x_1 while r' is aligned starting at position x_2.

Insertion:
When a read containing an insertion is aligned to the reference, the segments at either side of the read, r and r' (see Fig. 1b), are aligned to the reference while the insertion is clipped by the aligner. The length of the insertion can be found as the difference between the lengths of the clipped regions of the alignments ($\ell_{ins} = \ell_1 - \ell_2$). Consequently, the insertion can be inferred as the $(\ell_1 - \ell_2)$ bases abutting the reference position of the insertion. In practice, each read pair yields an insertion sequence. We use MAFFT (Katoh and Standley, 2013) to perform a multi-sequence alignment of these sequences and proceed to construct the consensus insertion sequence.

C.0.3. Indel detection from long reads.

Recall that we focus on indels with lengths up to 100bp; long reads readily span indels of such length. This, combined with the high rate of indel sequencing errors in long reads (frequently $> 10\%$), often causes the BWA-MEM aligner not to split the reads spanning the indel regions. Consequently, we rely on the CIGAR strings for each read obtained from the aligner to infer the positions and lengths of the indels. In particular, at each reference position x, we count the number of reads with an 'I', 'D' and 'S'/'D' at that position; these counts are denoted by c_I, c_D and c_C, respectively, while the number of reads bridging position x is denoted by c_x. An insertion is declared to have occurred at position x if $c_I + c_C > \alpha_{sig} c_x$, where α_{sig} is a user-defined threshold. Similarly, a deletion is declared to have occurred if $c_D + c_C > \alpha_{sig} c_x$. We iteratively merge the detected indels by pooling indels within m bases of each other. The indel length and positions are then determined as the average of the pooled indels's positions and lengths. The

consensus insertion sequence is found in a similar manner to the previously outlined procedure for handling short reads; the pooled insertion sequences are aligned using MAFFT before determining the consensus sequence of the requisite length. In all experiments, α_{sig} is set to 0.5 and m is set to 5.

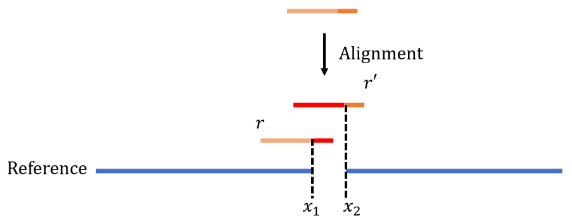

(a) Split alignments around a region with a deletion.

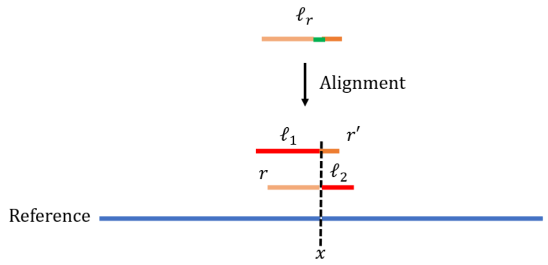

(b) Split alignments around a region with an insertion. The insertion is shown in green.

Fig. 1. Split alignments around regions with either an insertion or deletion. Clipped sections of the read alignments are shown in red, while the aligned sections are shown in orange.

C.1. XHap is highly accurate at detecting indels from short reads.

We demonstrate the efficacy of the outlined indel detection method by investigating its ability to detect indels of fixed lengths in experiments involving diploid genomes. To this end, we generate two sequences from the 10kbp reference genome specified in Section 3.1. A position along one of these sequences is selected at random for the indel placement. In case of an insertion, a random sequence of the given length ℓ is placed at this position; in the event of deletion, the next ℓ bases are removed from the selected sequence. The SNP calling is then performed as outlined in Section 3.1. We report results on the aforementioned metrics across 10 simulated datasets in Tables S2 and S3. Note that the proposed indel recovery methodology is not restricted to diploids; the diploid setting is selected for the ease of evaluation and demonstration.

Indel detection is characterized by the false negative rate and the errors in the position of the recovered indels. Additionally, the quality of a recovered insertion sequence is scored by aligning it to the true insertion and computing the fraction of bases correctly inferred. As shown in Table S2, the proposed methodology recovers intermediate-sized insertions nearly perfectly.

When evaluating performance of deletion discovery, we compare the length of the inferred deletion to the ground truth. Here, the proposed methodology performs well, achieving errors of less than 1 bp in the position of the recovered deletion and its length.

Table S2. Performance of insertion detection from short reads over 10 data instances. Performance is measured in terms of the false negative rate, position error and alignment score of the recovered insertion.

Length	False negative rate	Position error	Average alignment score
50	0	0 ± 1.1	0.968 ± 0.02
100	0	0 ± 0	0.990 ± 0.009
150	0.10	0.22 ± 0.63	0.990 ± 0.009

Table S3. Performance of deletion detection from short reads over 10 data instances. Performance is measured in terms of the false negative rate, position error and deviation in the length of the recovered deletion.

Length	False negative rate	Position error	Average length deviation
50	0.2	0.38 ± 1.58	0.88 ± 1.76
100	0	0.10 ± 0.30	0 ± 0.89
150	0	0.20 ± 0.60	0.20 ± 2.14

C.2. Indels are detected in spite of high sequencing errors in long reads

Long read sequencing platforms suffer from high indel sequencing errors (often $>$ 10%), so haplotype assembly and indel detection from these reads are inherently more challenging than with short reads despite the long reads spanning indels. In order to study the effectiveness of our proposed indel detection pipeline independent of the quality of the reconstructed haplotypes, we generate data using higher SNP rates than in Section 3.2; this enables highly accurate haplotype reconstruction and allows focusing on the indels. Specifically, we generate ground truth sequences as delineated in Section C.1, and then use PBSIM2 to generate sequencing reads.

As in the setting involving short reads, the proposed pipeline accurately locates both insertions are deletions with a mean error of less than 1 bp. However, the alignment scores for the recovered insertions, and the deviations in the detected lengths of the deletions, were significantly higher. This can be attributed to the high rate of indel sequencing errors inherent to the long read sequencing data.

Table S4. Performance of insertion detection from long reads over 10 data instances. Performance is measured in terms of the false negative rate, position error and the alignment score of the recovered insertion.

Length	False negative rate	Position error	Average alignment score
50	0	0.60 ± 0.92	0.844 ± 0.089
100	0	0.30 ± 0.78	0.798 ± 0.197
150	0	0 ± 3.32	0.795 ± 0.124

Table S5. Performance of deletion detection from long reads over 10 data instances. Performance is measured in terms of the false negative rate, position error and deviation in length of the recovered deletion.

Length	False negative rate	Position error	Average length deviation
50	0	1.20 ± 1.78	4.30 ± 4.80
100	0	0.40 ± 1.11	4.0 ± 5.16
150	0.1	0.89 ± 1.20	22.67 ± 20.78

References

K. Katoh and D. M. Standley. Mafft multiple sequence alignment software version 7: improvements in performance and usability. *Molecular biology and evolution*, 30(4): 772–780, 2013.

R. Poplin et al. Scaling accurate genetic variant discovery to tens of thousands of samples. *BioRxiv*, page 201178, 2017.
